# De Novo Assembly and Characterization of the *Xenocatantops brachycerus* Transcriptome

**DOI:** 10.3390/ijms19020520

**Published:** 2018-02-08

**Authors:** Le Zhao, Xinmei Zhang, Zhongying Qiu, Yuan Huang

**Affiliations:** 1 College of Life Sciences, Shaanxi Normal University, Xi’an 710062, Shaanxi, China; likezhaole@163.com (L.Z.); 13383242467@163.com (X.Z.); qiuzhongying11@126.com (Z.Q.); 2 School of Biological Sciences and Engineering, Shaanxi University of Technology, Hanzhong 723001, Shaanxi, China; 3 School of Basic Medical Sciences, Xi’an Medical University, Xi’an 710021, Shaanxi, China

**Keywords:** differentially expressed genes, different developmental stage, environmental adaptability, nutritional components and bioactive component

## Abstract

Grasshoppers are common pests but also have high nutritional and commercial potential. *Xenocatantops brachycerus* Willemse (Orthoptera: Acrididae) is an economically important grasshopper species that is reared in China. Using the IlluminaHiSeq^TM^ 4000 platform, three transcriptomes of the adult male, adult female, and nymph of *X. brachycerus* were sequenced. A total of 133,194,848 clean reads were obtained and de novo assembled into 43,187 unigenes with an average length of 964 bp (N50 of 1799 bp); of these, 24,717 (57.23%) unigenes matched known proteins. Based on these annotations, many putative transcripts related to *X. brachycerus* growth, development, environmental adaptability, and metabolism of nutritional components and bioactive components were identified. In addition, the expression profiles of all three transcriptome datasets were analyzed, and many differentially expressed genes were detected using RSEM and PossionDis. Unigenes. Unigenes with functions associated with growth and development exhibited higher transcript levels at the nymph stage, and unigenes associated with environmental adaptability showed increased transcription in the adults. These comprehensive *X. brachycerus* transcriptomic data will provide a useful molecular resource for gene prediction, molecular marker development, and studies on signaling pathways in this species and will serve as a reference for the efficient use of other grasshoppers.

## 1. Introduction

Grasshoppers (Orthoptera, Caelifera, Acridoidea) are widely distributed pests [1]. Plagues of grasshoppers can cause grassland degradation and agricultural disasters [2]. The growing use of chemical pesticides has led to the evolution of pesticide resistance and to severe negative effects on human health and the environment [3]. Hence, an interesting question is how to achieve an environmentally friendly management and utilization of grasshopper resources. Many researchers are seeking new methods to transform the harm caused by grasshopper resources into a benefit, and nutritional analyzes of many grasshopper species suggest that grasshoppers are an excellent source of most nutrients [4,5,6,7,8,9,10]. Because grasshoppers are rich in nutrients and easy to collect, most countries use grasshoppers as feed for birds, poultry, pigs, fish, and other animals consumed by humans [11,12]. In addition, some species of grasshoppers are commonly eaten in many countries, particularly in China, Japan, and Thailand [5,12,13,14]. Reports of genomic information (currently lacking) for prospective commercial grasshopper species seem timely and important to maximize the use of this environmentally friendly food source and to help understand the potential applications of grasshoppers as feed or food.

*Xenocatantops brachycerus* (Orthoptera: Acrididae) is both a pest and an economically important grasshopper species that is bred in Guangdong, China, as feed for pet birds and is also suitable for human consumption [15]. Recent research on *X. brachycerus* has mainly focused on its ecology and morphology [15,16]. Despite a recent report describing an assembly of the *X. brachycerus* mitogenome [17], other genetic investigations in this species are lacking.

High-throughput sequencing (HTS) technology has recently been employed to generate transcriptome data for non-model species and to provide valuable genome information, even in the absence of a genomic sequence [18]. Transcriptome data provide a useful perspective for the elucidation of gene functions, cell responses, and evolution, as well as of the molecular mechanisms of different biological processes [19]. To date, the transcriptomes of multiple orthopterans have been reported in the NCBI database, including those of *Locusta migratoria* [20], *Stenobothrus lineatus* [21], *Gryllus bimaculatus* [22], *Tetrix japonica* [23], *Shirakiacris shirakii* [24], and *Epacromius coerulipes* [25]. These transcriptomes have greatly enhanced the potential for postgenomic studies of orthopteran insects, however, no transcriptomic or genomic data are available for *X. brachycerus.* Currently, only 21 nucleotide sequences and 40 protein sequences from this species are available in the NCBI database (accessed on 20 September 2017).

In the present study, we performed de novo transcriptome sequencing of *X. brachycerus* for the first time using the Illumina HiSeq^TM^ 4000 sequencing platform. The transcriptomes of adult males, adult females, and nymphs were compared, and genes related to growth, development, environmental adaptability, and nutritional components and bioactive component metabolism were analyzed. The aim of this study was to analyze the transcriptome of *X. brachycerus* and produce a resource for large-scale gene discovery; this resource might facilitate studies of gene expression at different stages and in various biosynthetic pathways. Additionally, the data derived from this study will likely provide a basis for further investigations of the phylogenetic and speciation processes in *X. brachycerus*.

## 2. Results and Discussion

### 2.1. Sequencing Analysis and Assembly

The Illumina sequencing technology was used to sequence the transcriptome of *X. brachycerus* nymphs, adult females, and adult males. After filtering out adaptors and low-quality reads, 44,593,026, 44,314,184, and 44,287,638 clean reads were generated from *X. brachycerus* nymphs (XN), adult females (XF), and adult males (XM), respectively, with a Q30 percentage greater than 90%. The high Q30 value indicated that the sequencing data from the three *X. brachycerus* libraries were of high quality and suitable for generating assemblies.

The clean XN, XF, and XM reads were assembled into 32,653, 54,485, and 27,004 contigs, with mean contig sizes of 616 bp, 841 bp, and 625 bp, respectively (Table S1). Because a reference genome for *X. brachycerus* is not available, the contigs from all three libraries were combined and assembled into a reference unigene database using TGICL. This assembly yielded 43,187 unigenes for *X. brachycerus*, with an average length of 964 bp and an N50 of 1799 bp (File S1); subsequent sequence annotations were based on this database. Among these unigenes, 7166 (16.6%) were between 1000 bp and 2000 bp in size, 3300 (7.6%) were between 2000 and 3000 bp, and 2569 (5.9%) were longer than 3000 bp. The size distributions of all unigenes are shown in Table S2.

The completeness of the transcriptome assembly was assessed using Benchmarking Universal Single-Copy Orthologs (BUSCO) v3.0.2, and 93.4% of the BUSCO Arthropoda gene annotations were identified (Table S3). Based on these findings, we generated an ideal sequence assembly.

### 2.2. Functional Annotation

As shown in Table 1, 24,717 unigenes (57.2% of alldistinctunigenes) were successfully annotated in the *X. brachycerus* transcriptome, and the complete annotation results are presented in Table S4. According to the nrdatabase annotation, a high percentage of *X. brachycerus* sequences closely matched insect sequences (76.11%, 18,852). Among these genes, 51.52% mapped to *Zootermopsis nevadensis*, 5.76% mapped to *Tribolium castaneum*, and the remainder mapped to other insects. In addition, 1093 unigenes were annotated to proteins from Orthopteran species, such as *Locusta migratoria* (523), *Eyprepocnemisploransplorans* (164), and *Locusta migratoria manilensis* (49).

**Figure 1:**
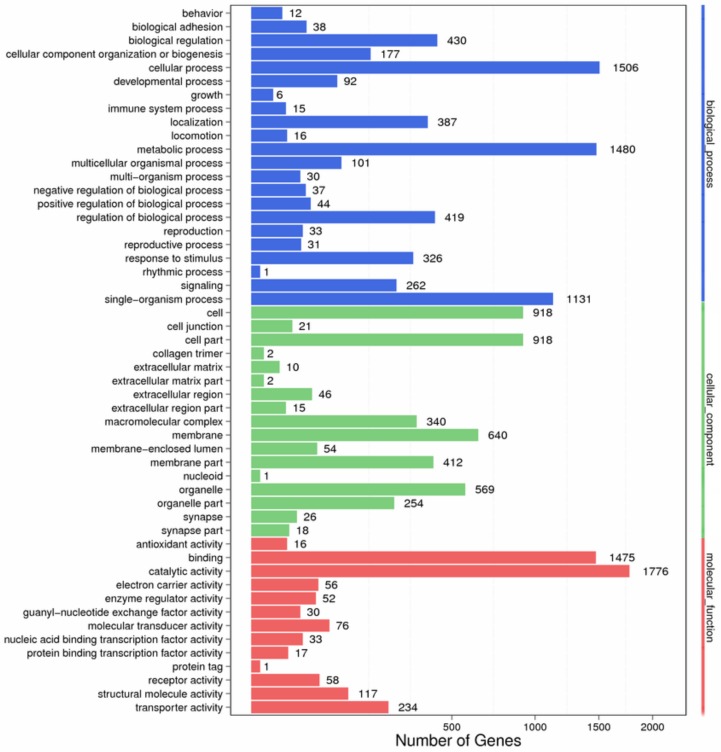
GO classifications of the assembled sequences from the *Xenocatantops brachycerus* transcriptome.

Based on the BLASTX annotation results, 3272 unigenes were classified into three main Gene Ontology (GO) categories and 52 subcategories. The main categories included biological processes (22 subcategories, 2088 unigenes), cellular components (17 subcategories, 1387 unigenes), and molecular functions (13 subcategories, 2779 unigenes) (Figure 1). For the biological process category, the most highly enriched GO terms were “cellular process” (1506 unigenes), “metabolic process” (1480 unigenes) and “single-organism process” (1131 unigenes). For cellular components, the most predominant GO terms were “cell” (918 unigenes), “cell part” (918 unigenes), and “membrane” (640 unigenes). For molecular functions, the most common GO terms were “catalytic activity” (1776 unigenes) and “binding” (1475 unigenes). The unigene distribution patterns among the three GO categories were similar to those observed for *Grapholita molesta* [26] and *Shirakiacris shirakii* [24]. Furthermore, a number ofunigenes were classified to some GO categoriesthat may contribute to tissue and organ morphogenesis in *X. brachycerus*, such as “structural constituent of chitin-based cuticle”, “chitin binding”, “mesoderm development”, “chitin metabolic process”, “tissue development”, and “structural constituent of the cuticle”.

**Figure 2:**
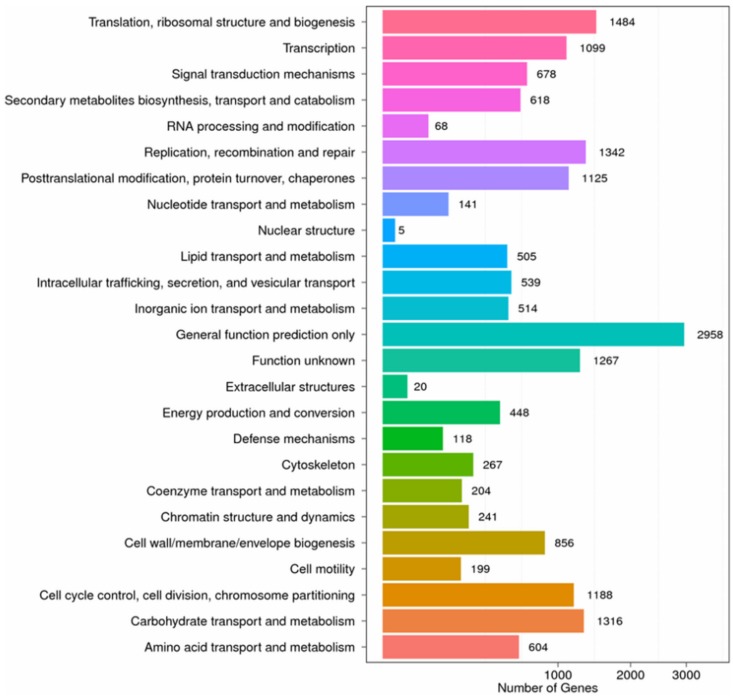
COG-based functional classification of the *Xenocatantops brachycerus* transcriptome.

The assembled unigenes were compared with the Clusters of Orthologous Groups (COG) database to predict and classify their possible functions and to further evaluate the completeness of the transcriptome and the effectiveness of the annotations. Nine thousand one hundred twenty-seven unigenes were classified into 25 COG categories (Figure 2). The categories of greatest interest in the present study were “secondary metabolites biosynthesis, transport, and catabolism” (618 unigenes), “lipid transport and metabolism” (505 unigenes), “defense mechanisms” (118 unigenes), and “amino acid transport and metabolism” (604 unigenes); the genes in these categories probably play significant roles in environmental adaptability and the metabolism of nutritional components and bioactive components.

**Figure 3:**
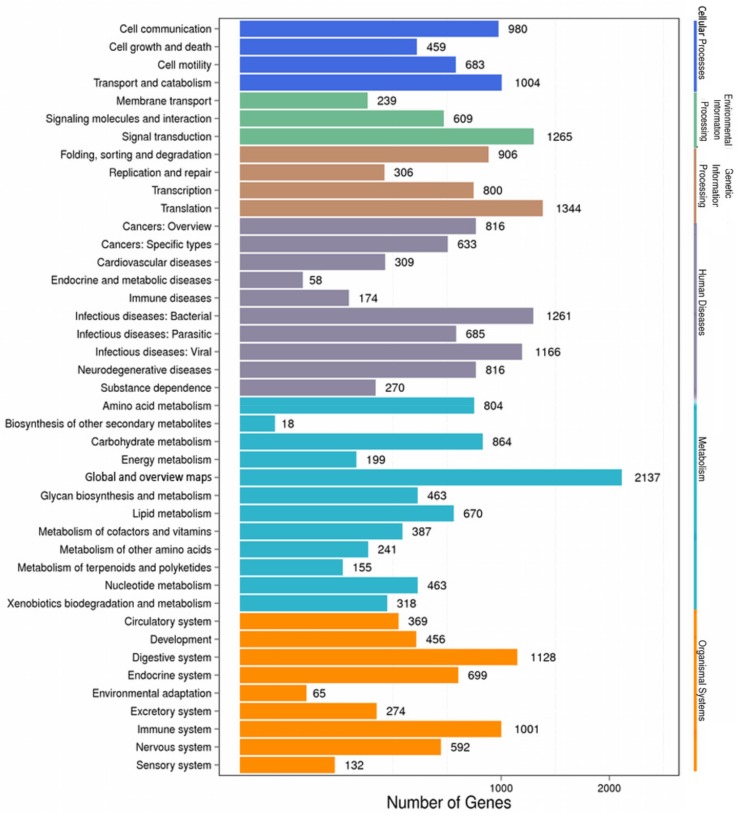
Categories of genes classified based on the Kyoto Encyclopedia of Genes and Genomes (KEGG) analysis.

A Kyoto Encyclopedia of Genes and Genomes (KEGG) analysis was used to explore biochemical pathways, including metabolic pathways and regulatory pathways, and identify active biological pathways in *X. brachycerus*. Ultimately, 15,762 unigenes were assigned to 4390 KEGG categories with six main clusters, including metabolism, genetic information processing, organismal systems, cellular processes, human diseases, and environmental information processing (Figure 3). Among these pathways, 318 unigenes were assigned to “xenobiotic biodegradation and metabolism”, which contained three major subcategories, all of which are related to pesticide degradation: “drug metabolism-cytochrome P450”, “drug metabolism of other enzymes”, and “metabolism of xenobiotics by cytochrome P450”. Additionally, large numbers of unigenes related to “development” (456 unigenes), “environmental adaptation” (65 unigenes), “immune system” (1001 unigenes), “lipid metabolism” (670 unigenes), and “biosynthesis of other secondary metabolites” (18 unigenes) were also identified. In addition, certain categories were annotated as related to human diseases, likely because of the large proportion of vertebrate sequences in the commonly used databases [23].

### 2.3. Functional Enrichment Analysis of DEGs

The clean reads from the three cDNA libraries were mapped on the reference database using Bowtie2 v2.3.1 software, and the mapping rates were 74.7%, 66.7%, and 77.7% for XF, XM, and XR, respectively. A fragments per kilobase per million (FPKM) analysis was performed to measure the gene expression levels (Table S5). The three samples were compared based on the calculated gene expression levels. We performed pair-wise comparisons of the gene expression levels in these three transcriptomes using the PossionDis algorithm, and a false discovery rate (FDR) ≤0.001 and a fold change ≥2.00 were used as the thresholds to screen the differentially expressed genes (DEGs) (Table S6). A total of 19,229 unigenes were differentially expressed between the three sample types. The results of the statistical analyzes of DEGs among the three *X. brachycerus* samples are presented in Figures S1 and S2. The largest differences were observed between XF and XN, in which 16,362 DEGs were identified, followed by XF and XM, with 15,351 DEGs, whereas XM and XN only exhibited 5932 DEGs.

Between the larval and adult stages, 2780 unigenes exhibited higher expression levels in the larval stage than in the adult stages (1375 in XF; 2545 in XM); these unigenes were assigned to 28 (19 in XF, 20 in XM, 11 in both) significantly enriched KEGG pathways and 203 (100 in XF, 150 in XM, 47 in both) significantly enriched GO terms (*p* ≤ 0.05). Among the encoded functional groups, the significantly enriched KEGG pathways were mainly associated with “central nervous system morphogenesis” (synaptic vesicle cycle, neuroactive ligand–receptor interaction, retrograde endocannabinoid signaling, GABAergic synapse, and circadian entrainment), “cellular proliferation” (adherens junction, phospholipase D signaling pathway, focal adhesion, and regulation of the actin cytoskeleton), and “phototransduction and tissue and organ development” (ECM–receptor interaction, phototransduction, hippo signaling pathway, and dorsoventral axis formation) (Table S7). Significantly enriched GO terms were also mainly involved in “tissue and organ morphogenesis” (e.g., structural constituent of chitin-based cuticle, chitin binding, mesoderm development, chitin metabolic process, tissue development, and structural constituent of the cuticle) (Table S7). The analyzes comparing the DEGs between the nymph and the adult stages provide the first broad outline of the mechanisms regulating *X. brachycerus* development and fundamental information for identifying candidate genes that might play critical roles in *X. brachycerus* development.

**Figure 4:**
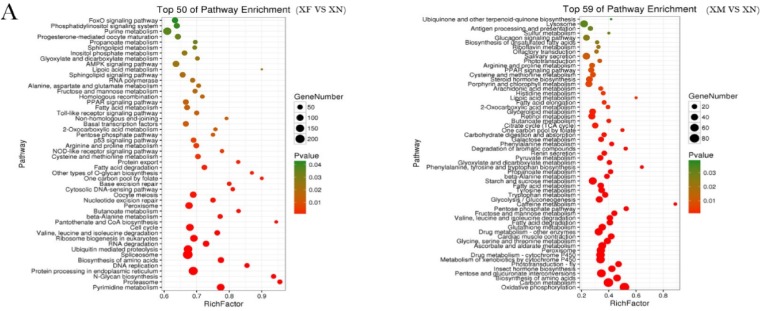

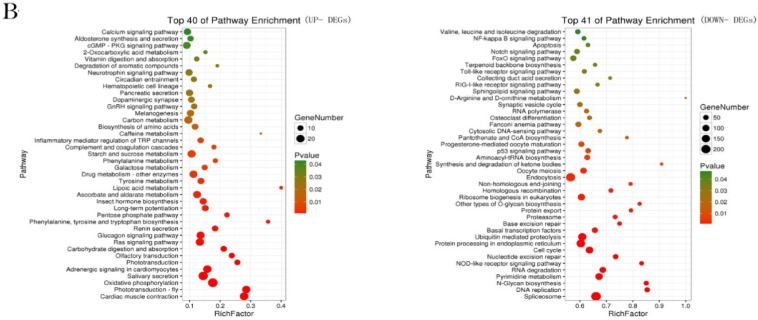
(**A**) Enrichment of metabolic pathways showing lower transcript levels in the larval stage than in the adult stages; (**B**) enrichment of metabolic pathways in female and male adults. The left plot corresponds to genes expressed at higher levels in males than in females and the right plot corresponds to genes expressed at lower levels in males than in females.

Notably, 15,970 DEGs were expressed at higher levels in the adult stages than in the nymph stage (14,987 in XF; 3387 in XM). These DEGs were assigned to 94 significantly enriched pathways (50 in XF, 59 in XM, 17 in both) (*p* ≤ 0.05) and 391 (157 in XF, 276 in XM, 40 in both) significantly enriched GO terms (*p* ≤ 0.05). Among the encoded functional groups, significantly enriched KEGG pathways and GO terms were mainly associated with environmental adaptability. For example, several significantly enriched pathways were related to defense responses or immune reactions, such as lysosomes, antigen processing and presentation, NOD-like receptor signaling, and Toll-like receptor signaling; in addition, several pathways were related to pesticide degradation, such as xenobiotic biodegradation and metabolism (drug metabolism-cytochrome P450, drug metabolism of other enzymes, and metabolism of xenobiotics by cytochrome P450) and “metabolism of terpenoids and polyketides” (insect hormone biosynthesis) (Figure 4A and Table S7). In this study, nymph and adult specimens of *X. brachycerus* were collected from the same environment, but a majority of the DEGs related to immune defense were expressed at higher levels in the adults than in the nymphs. According to several studies, immune defense is costly, and because resources are not limitless, organisms must allocate limited resources among competing, costly physiological functions [27,28,29]. The significantly enriched GO terms were mainly involved in aerobic respiration (e.g., ATP biosynthetic process, carbohydrate derivative metabolic process, mitochondrial proton-transporting ATP synthase complex, NADH dehydrogenase activity, oxidation–reduction process, and respiratory chain) (Table S7). Moreover, several significantly enriched pathways were related to metabolic pathways, including “amino acid metabolism” (e.g., tyrosine, tryptophan, histidine, phenylalanine, tyrosine, and tryptophan), “lipid metabolism” (arachidonic acid metabolism, biosynthesis of unsaturated fatty acids, fatty acid degradation, glycerolipid metabolism, sphingolipid metabolism, and steroid hormone biosynthesis), “cofactor and vitamin metabolism” (e.g., retinol metabolism, riboflavin metabolism, ubiquinone and other terpenoid-quinone biosynthesis, and pantothenate and CoA biosynthesis) (Figure 4A and Table S7). In addition, 2 and 13 DEGs involved in “flight behavior and adult locomotor behavior” and “olfactory transduction” were significantly enriched in the adults.

Regarding the comparison of the XF and XM stages, 1329 DEGs were upregulated in XM and were assigned to 40 significantly enriched pathways (*p* ≤ 0.05) and to 170 significantly enriched GO terms (*p* ≤ 0.05). The significantly enriched KEGG pathways were mainly associated with energy supply (galactose metabolism, pentose phosphate pathway, starch and sucrose metabolism, oxidative phosphorylation, and cardiac muscle contraction), the sensory system (phototransduction and olfactory transduction), and the digestive and excretory systems (salivary secretion, carbohydrate digestion and absorption, pancreatic secretion, vitamin digestion and absorption, and collection of duct acid secretion) (Figure 4B and Table S7).The significantly enriched GO terms were also mainly involved in energy supply (e.g., mitochondrial respiratory chain, aerobic respiration, carbohydrate metabolic process, ATP generation from ADP, and oxidation–reduction process) and ion transport (e.g., iron ion binding, metal ion transport, and calcium ion binding).

A total of 14,022 DEGs were expressed at higher levels in XF than in XM and were assigned to 41 significantly enriched pathways (*p* ≤ 0.05) and to 256 significantly enriched GO terms (*p* ≤ 0.05). The significantly enriched KEGG pathways were mainly associated with the transmission of genetic information, protein synthesis, the immune system (NOD-like receptor signaling pathway, RIG-I-like receptor signaling pathway, and Toll-like receptor signaling pathway), and “cell proliferation” (e.g., the spliceosome, DNA replication, ribosome biogenesis in eukaryotes, pyrimidine metabolism, nucleotide excision repair, and protein processing in the endoplasmic reticulum) (Figure 4B and Table S7). The significantly enriched GO terms were mainly involved in protein synthesis (e.g., protein transport, vesicle-mediated transport, and intracellular transport) and female germ cell development (e.g., female gamete generation, germ cell development, oocyte differentiation, and oogenesis). In addition, 100 and 9 DEGs related to oocyte development (e.g., mitotic-specific cyclin-B, the mitotic checkpoint serine/threonine-protein kinase BUB1, calmodulin-like protein 4 and sideroflexin-1) and sex determination (DNA methyltransferase, sex lethal, and transformer-2) showed increased transcription in females (Table S8). Sxland Tra2 are both associated with sex determination in *Drosophila* [30,31,32], and, in the present study, transcripts for both of these putative DEGs were expressed at higher levels in females than in males.

### 2.4. Validation of RNA-Seq Gene Expression Data

**Figure 5:**
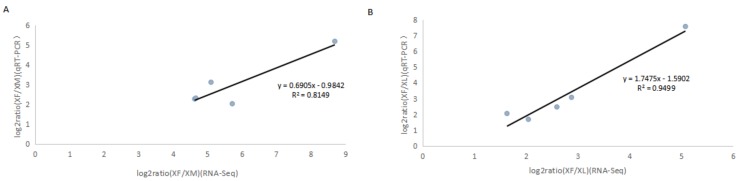
Expression levels of DEGs in *Xenocatantops brachycerus.* (**A**) Transcriptional variation of five genes between XL and XF, *R*^2^ = 0.8149 indicates a very significant correlation between qRT-PCR and RNA-Seq data; (**B**) transcriptional variation of five genes between XM and XF. *R*^2^ = 0.9499 indicates a very significant correlation between qRT-PCR and RNA-Seq data.

We performed qRT-PCR on five selected sex determination-related genes or DEGs with particularly low *p*-values to confirm the reliability of the RNA-Seq data. We designed primers for those genes and used β-actin as a control to measure and compare their expression levels in XM and XN, as well as in XM and XF. These DEGs were putative tra2, dnmt1.1, dnmt2, RNA-binding protein (RBP1), and Sxl transcripts. Overall, consistent expression patterns for these five genes were obtained between the qRT-PCR and RNA-Seq analyzes (Figure 5 and Figure S3), indicating that the transcriptome sequencing results were reliable and would enable us to make reasonable deductions based on the functional enrichment analysis of the DEGs.

### 2.5. Candidate Genes Involved in Growth, Development, Immunity, and Nutritional and Bioactive Compounds Metabolism

Many gene sequences that are putatively involved in growth and development were annotated in the *X. brachycerus* transcriptome. In insects, juvenile hormones (JHs) and ecdysone play central roles in controlling many important aspects of growth and development, such as the regulation of the timing and nature of insect moults, the induction and maintenance of diapause, the foraging behavior, the determination of body color and flight activity [33,34]. In the present study, 114 and 52 putative genes encoded enzymes and proteins involved in the metabolism, transport, or signal transduction of JHs and ecdysone, respectively (Table S9). We identified 588 *X. brachycerus* genes (Table S10) that were associated with the KEGG pathways Wnt (ko04310), Notch (ko04330), TGF-β (ko04350), JAK/STAT (ko04630), MAPK-fly (ko04013), and Hedgehog (ko04340). In addition, all members of the Hedgehog pathway were identified. These pathways play critical roles in insect growth and development [35]. For example, the Wnt signaling pathway has been shown to coordinate crucial cellular processes, such as the control of axis elongation and leg development in one short-germ insect [36], and the Notch signaling pathway is involved in hormone-dependent pattern formation in butterfly nymphs [37]. In addition, the components of these six signaling pathways are highly evolutionarily conserved among insect species [38].

We analyzed transcripts related to immune responses, stress responses, and detoxification mechanisms. We obtained a large number of transcripts related to immune mechanisms. In insects, non-self-recognition is the initial process in the innate immune response [39]. The recognition of prokaryotic pathogens is mediated by pattern recognition proteins (PRPs) such as peptidoglycan recognition protein (PGRP) and C-type lectin (CTL) [40,41]. In invertebrates, PRPs are important components of humoral innate immune responses, which enable the host to effectively resist pathogenic invasions by specifically recognizing sugars on the surface of microorganisms [40]. In the present study, 14 putative PGRP genes and 31 putative CTL genes were annotated (Table S11). Three signal transduction pathways were also identified: the Toll-like receptor (TLR) (ko04620), NOD-like receptor (ko04621), and RIG-I-like receptor (ko04622) pathways. These pathways play crucial roles in activating immune defense mechanisms [42]. For example, the TLR pathway is associated with the *Drosophila* 18-wheeler protein, a transmembrane protein proposed to sense antibacterial infections and generate innate immune responses [43]. Here, 125 *X. brachycerus* genes related to these three pathways were identified (Table S12). Additionally, we obtained 389 putative transcripts related to detoxification mechanisms, and these transcripts were highly enriched in the pathways “metabolism of xenobiotics by cytochrome P450” (ko00980), “drug metabolism-cytochrome P450” (ko00982), “drug metabolism of other enzymes” (ko00983), and “glutathione metabolism” (ko00480). These sequences mainly encode members of three major multigene enzyme families: the cytochrome P450s, carboxylesterases (CXEs), and glutathione S-transferases (GSTs) (Table S13). In insects, cytochrome P450s, CXEs, and GSTs play essential roles not only in the metabolism of a variety of physiologically important endogenous compounds, but also in the detoxification of various harmful exogenous compounds, such as plant allelochemicals and insecticides [44].

Grasshoppers are a good source of nutritional and bioactive compounds (e.g., chitin). Many studies have established the physicochemical characteristics of the nutrient composition, amino acid profile, lipid composition, vitamin content, and bioactive compounds of grasshoppers, but the corresponding genetic information available in this field is limited [3,7,8,9]. Grasshoppers contain high-quality protein because of the presence of all essential amino acids in the recommended ratios, and the essential amino acid content of grasshoppers is comparable to that of resource-intensive animal protein sources [45,46]. We obtained 805 transcripts putatively associated with 15 amino acids: eight essential amino acids (valine, isoleucine, leucine, lysine, threonine, methionine, phenylalanine, and histidine), one conditionally essential amino acid (tyrosine) and six nonessential amino acids (arginine, glutamic acid, serine, glycine, alanine, and proline) in the *X. brachycerus* transcriptome (Table S14). Grasshoppers also contain large amounts of vitamins. For example, vitamin B_2_ content of *Oxya chinensis* is three times higher than that of eggs, and its vitamin E content is five times higher than that of pork [9]. KEGG pathways related to thiamine (ko00730), riboflavin (ko00740), biotin (ko00780), folate (ko00790), retinol (ko00830), and vitamin B6 (ko00750) metabolic pathways were identified in this study, and 299 genes were related to these pathways (Table S14). Although grasshoppers are mainly considered a protein source, they are also rich in essential fatty acids (EFAs), such as linoleic acid, α-linolenic acid, arachidonic acid, and phospholipids [8,47,48]. In most cases, linoleic acid and α-linolenic acid are among the essential fatty acids present in the highest proportions in grasshoppers [48]. Phospholipids are major building blocks of life; their in vivo synthesis requires multiple enzymes and cofactors, and some phospholipids are most likely conditionally essential nutrients [49]. Phospholipid mixtures (lecithin) were among the first recognized health foods, and they are widely used, highly functional, standard additives to food [49,50]. In general, the phospholipid content of insects ranges from 0.4% to 3.3%, which is significant for industrial lecithin production [3,47,48]. The phospholipids extracted from insects have been used to produce food additives that provide additional physiological benefits beyond simply meeting basic nutritional needs, and in medicines, cosmetics, and other industries [3,9]. In the present study, KEGG-annotated transcripts involved in essential fatty acid metabolism (ko00590, ko00591, and ko00952) were detected, and 275 transcripts were putatively related to functional fatty acids (Table S14). Moreover, compared with other food and feed sources, insects exhibit high fiber contents (ranging from 4 to 11%), making them a nutritionally balanced foodstuff [45]. Chitin is a carbohydrate polymer and an important bioactive component in insects. Chitin from the shells of lobsters, crabs, and crayfish has been approved for use in cereals as a source of fiber and calcium in Japan, on the basis of previous research, and insects also represent a potential source of fiber because of their high chitin content, accounting for approximately 10% of a whole dried insect [46]. We identified 118 unigene transcripts putatively related to chitin in the *X. brachycerus* transcriptome (Table S14). If protein concentrates from dechitinized grasshoppers become acceptable for consumption and are produced at a large scale, the chitin byproduct may be a substantially valuable fiber source, as suggested by De Foliart (1992) [46]. Moreover, explorations of the molecular mechanisms underlying the potential uses of the nutritional components and bioactive compounds of *X. brachycerus* and the development of procedures for the commercial rearing of this species would be interesting.

## 3. Materials and Methods

### 3.1. Ethics Statement

Our research did not involve human participants or samples, and no specific permits were required for this field study.

### 3.2. Species Collection, RNA Extraction, and Illumina Sequencing

All *X. brachycerus* specimens were collected from Mount Wutai (34.0287° N, 108.9698° E) in Xi’an, China on 12 September 2015. Grasshoppers were classified into nymph samples (XN: from third instar to fourth instar), adult female samples (XF), and adult male samples (XM). The whole body, excluding the gut, was collected from 15 grasshoppers (5 from XN, 5 from XF, and 5 from XM) and stored in liquid nitrogen until RNA isolation. Total RNA was extracted from each individual using TRIzol reagent (Invitrogen, Carlsbad, CA, USA), according to the manufacturer’s protocol. Afterwards, the purity and integrity of RNA were assessed using a Nano Drop 2000 (Thermo Scientific, Wilmington, DE, USA) and an Agilent 2100 Bioanalyzer (Agilent Technologies, Mississauga, ON, Canada) with a minimum integrity number value of 7. Equal amounts of the RNA samples from XN, XF, and XM were then pooled together for cDNA synthesis and sequencing. RNA-Seq library preparation and sequencing were performed by BGI (Shenzhen, China). Three cDNA libraries were prepared using the TruSeq^TM^ RNA Sample Prep Kit v2 (Illumina, Inc., San Diego, CA, USA). An Agilent 2100 Bioanaylzer and ABI StepOnePlus Real-Time PCR System were used for quantification and qualification of these libraries. Finally, the three transcriptome libraries were sequenced using paired-end IlluminaHiSeq 4000 technology (Illumina Inc., San Diego, CA, USA), producing 100 bp paired-end reads.

### 3.3. De Novo Assembly and Annotation

For projects without reference genomes, clean reads must be assembled after sequencing to produce a reference sequence for subsequent analysis. The raw reads were cleaned using filter fq (BGI in-house script), as follows: (1) reads with adaptors were removed; (2) reads in which unknown bases (N) comprised more than 5% of the sequence were removed; (3) low-quality reads (defined here as containing more than 20% of bases with a quality of less than 10) were removed. After filtering, the remaining reads were called “clean reads” and stored in FASTQ format. Additionally, the Q20, Q30, GC content, and repetitive sequence values were calculated, and all downstream analyzes were performed using high-quality clean data (sequences with a minimum PHRED score of Q30, or 99.9% accuracy).

High-quality clean reads were de novo assembled using Trinity v2.0.6 software [51] without a reference genome (parameter settings: --min_contig_length 150 --CPU 8 --min_kmer_cov 3 --min_glue 3 --bfly_opts ‘-V 5 --edge-thr=0.1 –stderr’). The Trinity assembly results were called transcripts, and then gene family clustering was performed using TGICL clustering software v2.0.6 [52] to obtain the final unigenes. The parameters of TGICL used in the present study were: -l 40 -c 10 -v 25 -O ‘-repeat_stringency 0.95 -minmatch 35 -minscore 35’. The unigenes were divided into two classes: clusters (CL), composed of several unigenes with a shared similarity greater than 70%, and singletons (unigenes). Furthermore, the completeness of the transcriptome assembly of *X. brachycerus* was assessed using BUSCO v3.0.2 [53]. The closest representative model to insects is fly (*Drosophila*), and the database “arthropoda_odb9” was chosen as a lineage-specific profile library. The raw sequence reads and assembly data were submitted to the European Nucleotide Archive database (ENA; Accession ID: PRJEB24040) and the China National Genebank (CNSA; Accession ID: CNP0000019).

All assembled sequences were aligned to the NCBI non-redundant protein sequence (nr) and nucleotide sequence (nt) databases, a manually annotated and reviewed protein sequence database (Swiss-Prot), the KEGG database, and the COG database using BLAST v2.2.23 (*e*-value ≤ 10^–5^). The best hits from these databases were employed to determine the sequence orientations and coding sequences (CDSs) of the unigenes. ESTScanv3.0.2 was used to predict the direction of the sequences and CDSs when a unigene was not aligned in any of the databases [54]. Blast2GO v2.5.0 was used to determine the GO annotations for unigenes annotated with the nr database [55], and WEGO v2.0 software [56] was used to determine GO functional classifications and assess the distribution of unigene functions. Finally, InterProScan5 v5.11-51.0 was used to analyze protein functions by classifying the proteins into families and predicting domains and important sites [57].

### 3.4. Differentially Expressed Unigenes (DEGs)

The clean reads from each sample were compared with the reference unigene database using Bowtie2 v2.1.0 software [58] (parameter settings: -q --phred64 --sensitive --dpad 0 --gbar 99999999 --mp 1,1 --np 1 --score-min L,0,-0.1 -I 1 -X 1000 --no-mixed --no-discordant -p 1 -k 200). The unigene expression levels were then estimated using RSEM v1.2.12 [59] with the default parameters for each sample, employing FPKM values. DEGs between groups (XF versus XM, XM versus XN, and XF versus XN) were analyzed using the PoissonDis algorithm [60,61,62], which is based on the Poisson distribution, performed as described by Audic [63]. The FDR is a statistical measure used to determine the threshold for *p*-values in multiple tests [64]. Fold changes (log2 ratio) were estimated according to the normalized gene expression level in each sample [65]. For screening purposes, an FDR ≤0.001 and a fold change ≥2.00 were used as the thresholds for identifying significant DEGs. DEGs were then mapped to the GO terms and the KEGG databases for functional and pathway enrichment analyzes (corrected *p* ≤ 0.05).

### 3.5. Quantitative Real-Time RT-PCR (qRT-PCR)

Five genes showing high levels of significance or important sex determination-related genes were selected for qRT-PCR analysis with β-actin as the reference gene to validate the RNA-Seq results. Specific primers for the candidate genes were designed using Primer Premier 5.0 (Premier Biosoft International, Palo Alto, CA, USA) (Table S15). Total RNA was extracted from the XN, XF, and XM specimens, and the RNA levels were measured with the SYBR PremixExTaq quantitative PCR kit (TaKaRa, Dalian, China). Then, qRT-PCR was performed using a PrimeScript^TM^ RT reagent qPCR Kit (TaKaRa) and the following parameters: 95 °C for 30 s, and 40 cycles at 95 °C for 15 s and at 60 °C for 34 s. Fluorescence intensity was measured using CFX96 (Bio-Rad, Hercules, CA, USA). Triplicates of each reaction were performed. β-actin was simultaneously used as an endogenous control. The expression of the target gene relative to β-actin was determined using the formula 2^−ΔΔ*C*t^ [66]. The experiment was repeated three times for each group, and mean values were calculated. All data are presented as the mean ± SD. Pearson’s correlation coefficient (PE) and significant differences between samples were obtained using SPSS v16.0 software.

## 4. Conclusions

In this study, we provide the first report of the *X. brachycerus* transcriptomes at different development stages using the Illumina HiSeq4000 platform. A large number of transcripts and annotation resources were developed and may prove to be valuable tools for future genetic and genomic studies of *X. brachycerus* and other related grasshopper species. Moreover, this transcriptome assembly may also be used as a reference for studies comparing the biology within families or genera of Orthoptera.

## Figures and Tables

**Table 1:** Functional annotations of the *Xenocatantops brachycerus* transcriptome.

Item	Number	Percentage
Total	43,187	100%
nr-annotated	21,978	50.89%
nt-annotated	12,071	27.95%
Swiss-Prot-annotated	17,692	40.97%
KEGG-annotated	15,762	36.50%
COG-annotated	9127	21.13%
InterPro-annotated	15,099	34.96%
GO-annotated	3272	7.58%
All annotated	24,717	57.23%

## Supplementary Materials

The following are available online at http://www.mdpi.com/1422-0067/19/2/520/s1.
