# Supplementary material for: De Novo Assembly and Characterization of the Xenocatantops brachycerus Transcriptome
Source: Int J Mol Sci. 2018 Feb 8;19(2):520. doi: 10.3390/ijms19020520 (PMC5855742; doi:10.3390/ijms19020520)
Supplement: Supplementary file 1 [file ijms-19-00520-s001.zip › supplementary file/captions.docx]

**File S1.** Complete sequence assembly.

**Table S1.** Sequencing output statistics.

**Table S2.** Distribution of unigene lengths.

**Table S3.** Summarized benchmarks in the BUSCO notation.

**Table S4.** Complete annotation results.

**Table S5.** Summary of unigene expression levels.

**Table S6.** Statistics for DEGs between the three sample types.

**Table S7****.** Statistics for significantly enriched pathways and GO terms between the nymph and adult stages.

**Table S8.** Annotation ofoocyte- and sex determination-related candidate genes in the *X. brachycerus* transcriptome.

**Table S9.** Annotation of JH- and ecdysone-related candidate genes in the *X. brachycerus* transcriptome.

**Table S10.** Candidate genes in the *X. brachycerus* transcriptome involved in KEGG pathways related to growth and development.

**Table S11.** Annotation of candidate peptidoglycan recognition protein (PGRP)- and C-type lectin (CTL)-related genes in the *X. brachycerus* transcriptome.

**Table S12.** Candidate genes in immunity-related KEGG pathways in the *X. brachycerus* transcriptome.

**Table S13.** Candidate genes in pesticide metabolism-related KEGG pathways in the *X. brachycerus* transcriptome. Table S14. Annotation of candidate nutritional and bioactive component-related genes in the *X. brachycerus* transcriptome. Table S15: Primers used for qRT-PCR.

**Figure S1.** Venn diagram of expressed genes among the three different *X. brachycerus* transcriptomes.

**Figure S2.** Hierarchical clustering-based expression patterns of DEGs. The *X*-axis represents the results from the hierarchical clustering analysis of the differences, the *Y*-axis represents the DEGs, and the colours represent the expression levels. The blue area represents down-regulated genes, and the red area represents up-regulated genes.

**Figure S3.** Expression levels of DEGs in *Xenocatantops brachycerus.* (A) Comparative gene expression analysis based on qRT-PCR and RNA-Seq data from *X. brachycerus* between XF and XM. *X*-axis: the 5 DEGs; *Y*-axis: relative expression level of each unigene; * indicates significantly different expression (*p* < 0.05); (B) Comparative gene expression analysis based on the qRT-PCR and RNA-Seq data from *X. brachycerus* between XF and XN. *X*-axis: the 5 DEGs; *Y*-axis: relative expression level of each unigene. * Indicates significantly different expression (*p* < 0.05).
