# Supplementary figures and images for: De Novo Assembly and Characterization of the Xenocatantops brachycerus Transcriptome
Source: Int J Mol Sci. 2018 Feb 8;19(2):520. doi: 10.3390/ijms19020520 (PMC5855742; doi:10.3390/ijms19020520)

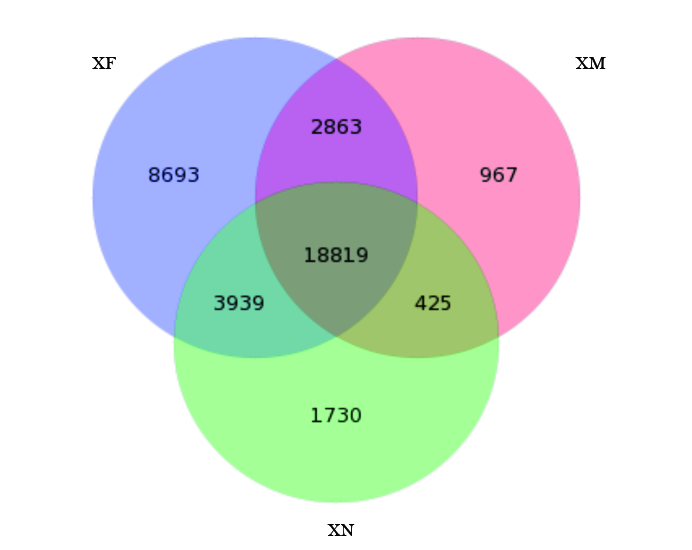

Supplement: Supplementary file 1 [file ijms-19-00520-s001.zip › supplementary file/Figure S1.tif]

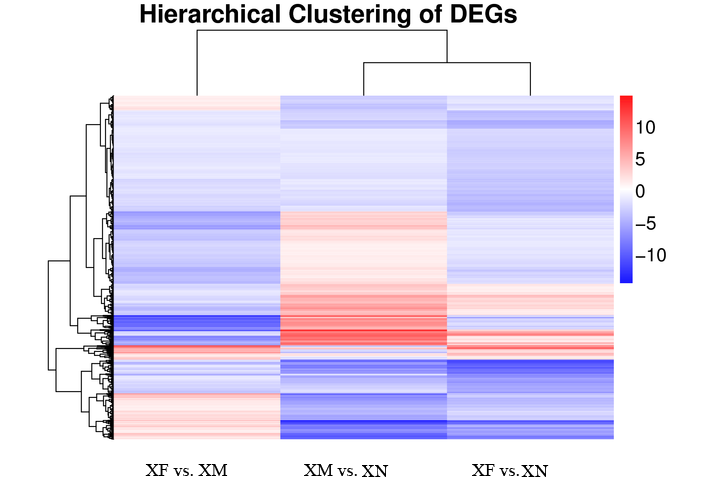

Supplement: Supplementary file 1 [file ijms-19-00520-s001.zip › supplementary file/Figure S2.tif]

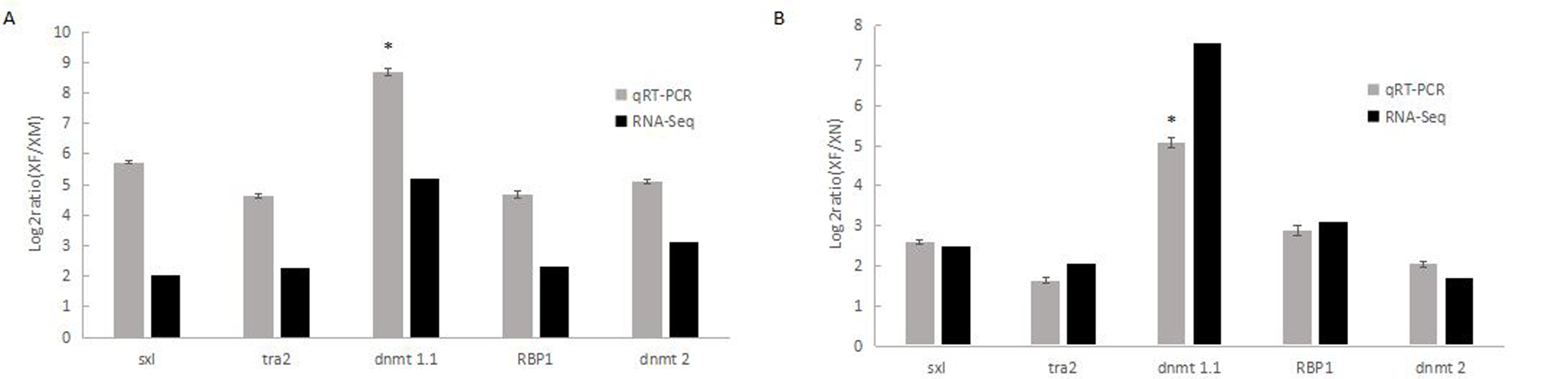

Supplement: Supplementary file 1 [file ijms-19-00520-s001.zip › supplementary file/Figure S3.tif]
